# Distinct Immune Response in Two MERS-CoV-Infected Patients: Can We Go from Bench to Bedside?

**DOI:** 10.1371/journal.pone.0088716

**Published:** 2014-02-14

**Authors:** Emmanuel Faure, Julien Poissy, Anne Goffard, Clement Fournier, Eric Kipnis, Marie Titecat, Perinne Bortolotti, Laura Martinez, Sylvain Dubucquoi, Rodrigue Dessein, Philippe Gosset, Daniel Mathieu, Benoit Guery

**Affiliations:** 1 Host-Pathogen translational research group, Université de Lille 2, Lille, France; 2 Pôle de Réanimation, Hôpital Roger Salengro, Centre Hospitalier Régional et Universitaire de Lille, Université de Lille 2, Lille, France; 3 Laboratoire de Virologie, Centre de Biologie Pathologie, Centre Hospitalier Régional et Universitaire de Lille, Université de Lille 2, Lille, France; 4 Clinique des Maladies Respiratoires, Endoscopie bronchique et pleurale - Endoscopie Interventionnelle, Centre Hospitalier Régional et Universitaire de Lille, Université de Lille 2, Lille, France; 5 Institut de Microbiologie, Centre Biologie Pathologie, Centre Hospitalier Régional et Universitaire de Lille, Université de Lille 2, Lille, France; 6 Institut d'Immunologie, Centre Biologie Pathologie, Centre Hospitalier Régional et Universitaire de Lille, Université de Lille 2, Lille Cedex, France; 7 Centre National de la Recherche Scientifique, Unité Mixte de Recherche 8204, Lille, France; Institut National de la Santé et de la Recherche Médicale, U1019, Lille, France; Fondazione IRCCS Policlinico San Matteo, Italy

## Abstract

One year after the occurrence of the first case of infection by the Middle East Respiratory Syndrome coronavirus (MERS-CoV) there is no clear consensus on the best treatment to propose. The World Health Organization, as well as several other national agencies, are still working on different clinical approaches to implement the most relevant treatment in MERS-CoV infection. We compared innate and adaptive immune responses of two patients infected with MERS-CoV to understand the underlying mechanisms involved in the response and propose potential therapeutic approaches. Broncho-alveolar lavage (BAL) of the first week and sera of the first month from the two patients were used in this study. Quantitative polymerase chain reaction (qRTPCR) was performed after extraction of RNA from BAL cells of MERS-CoV infected patients and control patients. BAL supernatants and sera were used to assess cytokines and chemokines secretion by enzyme-linked immunosorbent assay. The first patient died rapidly after 3 weeks in the intensive care unit, the second patient still recovers from infection. The patient with a poor outcome (patient 1), compared to patient 2, did not promote type-1 Interferon (IFN), and particularly IFNα, in response to double stranded RNA (dsRNA) from MERS-CoV. The absence of IFNα, known to promote antigen presentation in response to viruses, impairs the development of a robust antiviral adaptive Th-1 immune response. This response is mediated by IL-12 and IFNγ that decreases viral clearance; levels of both of these mediators were decreased in patient 1. Finally, we confirm previous *in vitro* findings that MERS-CoV can drive IL-17 production in humans. Host recognition of viral dsRNA determines outcome in the early stage of MERS-CoV infection. We highlight the critical role of IFNα in this initial stage to orchestrate a robust immune response and bring substantial arguments for the indication of early IFNα treatment during MERS-CoV infection.

## Introduction

Coronaviruses are large enveloped single-stranded RNA viruses associated with a wide range of clinical presentations in animals and humans[1]. In September 2012, a novel human coronavirus, named Middle East Respiratory Syndrome Coronavirus (MERS-CoV), belonging to lineage C of the genus *Betacoronavirus*, was identified in two patients with severe acute respiratory disease in Saudi Arabia [2]. Several countries (France, Germany, Jordan, Qatar, Saudi Arabia, Tunisia, and the United-Kingdom) reported new cases. Recently, Assiri *et al.* published an extensive description of 47 cases from Saoudi Arabia [3].

Coronaviruses, like most successful viruses, have evolved strategies to evade the innate immune response. Type-1 interferon (IFN) expression is a crucial component of this initial response, and coronaviruses have developed strategies to prevent IFN induction[4]. Double-stranded RNA (dsRNA) are recognized by the innate immune system through activation of cytosolic (RIG-1 and MDA-5)[5] and membranous (TLR3)[6], [7] pattern recognition receptors (PRRs). The recognition by these PRRs triggers the activation of Interferon Regulatory Factor (IRF3, IRF7) which leads to the induction of type-1 interferon, such as IFNα and IFNβ[8]. Type-1 IFN activates immune anti-viral effectors such as Natural Killer (NK) cells[9], T CD8+ cells and macrophages, allowing viral clearance[10]. Consistent with the studies demonstrating that Severe Acute Respiratory Syndrome (SARS)-CoV proteins contribute to dampen type-1 IFN signalling to evade innate immunity and viral clearance[1], [7], an *in vitro* study reported a beneficial effect of Interferon-alpha (IFNα) treatment on MERS-CoV replication in a cellular model[11]. In this study the authors showed that MERS-CoV was 50-100 times more sensitive to IFN-α treatment than SARS-CoV. However, to date there is no clear therapeutic protocol beside supportive treatment in MERS-CoV infection.

Here we report the analysis of the immune response in two concomitant cases of patients infected with MERS-CoV admitted to the Lille University Teaching Hospital between April and June 2013. The clinical presentation was previously reported [12], and the outcome of these two patients largely differed. Indeed, the first patient died after one month in the intensive care unit, while the second patient is still recovering from infection. We compared the early immune response of these two patients to describe putative underlying mechanisms of MERS-CoV infection and propose potential therapeutic targets.

## Materials and Methods

### Samples

#### Broncho-alveolar lavage (BAL) samples

We obtained samples from BAL performed during the first week after the onset of symptoms for the two infected patients: patient 1 (n = 2, duplicates) and patient 2 (n = 2, duplicates). Control samples (n = 4) were obtained from patients from the Pulmonology department requiring BAL for reasons other than respiratory tract infection. Additional control samples for bacterial respiratory tract infection were obtained from patients with *Streptococcus pneumoniae*-induced pneumonia requiring BAL in the Infectious Diseases Unit (n = 2). Finally, during recovery, patient 2 presented a Herpes Simplex Virus type 1 (HSV) lung replication related to a compensatory anti-inflammatory response syndrome (CARS syndrome) (n = 1, duplicates), a BAL was performed for diagnosis and used in this study as control for virus-induced pneumonia. BAL supernatants were used for enzyme-linked immunosorbent assay (ELISA), BAL cells were used for RNA extraction and quantitative polymerase chain reaction (qRT-PCR).

#### Serum samples

Serum samples were obtained from patient 1 and 2. Control samples were obtained from the serological biobank of the Institute of Immunology (graciously provided by Sylvain Dubucquoi M.D. PhD). Samples from a renal transplant recipient with a comparable immunosuppressive therapy were used as control for patient 1. Patient 2 had an immunological analysis performed prior to MERS-CoV infection in November 2012 in the context of his histamine-induced angioedema. These biobanked samples were used in this study as controls.

#### qRT-PCR expression analysis

Samples were kept at −80°C. RNA extractions of were performed using Qiagen RNeasy mini kit (Qiagen, UK). Isolated RNA were reverse-transcribed with the High Capacity cDNA Reverse Transcription Kit (Life technologies, UK) according to the manufacturer's instructions. The resulting cDNA (equivalent to 25 ng of total RNA) was amplified using the KAPASYBR real-time PCR kit (Kapa Biosystems, United States of America) and detected on an ABI7900HT (Applied Biosystem, UK). RT-PCR was performed with the following forward and reverse primers for: GAPDH (5′-ACCCACTCCTCCACCTTTGA -3′) and (3′-CATACCAGGAAATGA GCTTGACAA-5′), RIG-1 (5′-GCCATTACACTGTGCTTGGAGA-3′) and (3′- CCAGTTGCAATATCCTCCACCA-5′), MDA-5 (5′- TGTATTCATTATGCTACAGAACTG-3′) and (3′- ACTGAGACTGGTACTTTGGATTCT-5′), IRF3 (5′-AGGACCCTCACGACCCACATAA -3′) and (3′-GGCCAACACCATGTTACCCAGT-5′), IRF7 (5′- TGGTCCTGGTGAAGCTGGAA-3′) and (3′- GATGTCGTCATAGAGGCTGTTGG-5′), IFNα (5′-AGCCATCTCTGTCCTCCATGAG-3′) and (3′- TGCATCACACAGGCTTCCAA-5′) IFNβ (5′- GTCTCCTCCAAATTGCTCTC-3′) and (3′- ACAGGAGCTTCTGACACTGA-5′), IL-17A (5′-ACTACAACCGATCCACCTCAC-3′) and (3′-ACTTTGCCTCCCAGATCACAG-5′). On completion of the PCR amplification, a DNA melting curve analysis was carried out in order to confirm the presence of a single amplicon. GAPDH was used as an internal reference gene in order to normalize the transcript levels. Relative mRNA levels (2-ΔΔCt) were determined by comparing (a) the PCR cycle thresholds (Ct) for the gene of interest and GAPDH (ΔCt) and (b) ΔCt values for treated and control groups (ΔΔCt).

#### Cytokine Assay

Cytokines levels in BAL supernatants and sera were assessed by ELISA. Human Duoset kits (R&D Systems, UK) for CXCL10 or Human Ready-to-go kit (eBiosciences, USA) for IL-12, IL-10, IL-17, IL-23, IFNα and IFNγ.

### Statistical analysis

Statistical analysis was performed using Prism 6 software (GraphPad).

Kruskall-Wallis test was used for all comparisons except when otherwise indicated. Significance was accepted at p<0.05.

### Ethics Statement

We obtained the approval of the Lille Hospital Ethic committee. All the participants provided their written or verbal conformed to participate on this study. The consent procedure was approved by the Ethic committee. The patients in this manuscript or their next of kin have given written informed consent or verbal consent, when writing was not physically possible, to publication of their case details.

## Results

Patient 1, a 64-year-old man, had undergone renal transplantation in 1998 for end-stage renal failure secondary to diabetes. His treatments associated mycophenolate mofetil, ciclosporin, and prednisone. Clinical symptoms began on April 22; he was transferred to the intensive care unit and intubated on April 30. On May 8, the patient required implementation of extracorporeal membrane oxygenation (ECMO) and died on May 28 of refractory multiple organ failure.

Patient 2, a 51-year-old man, was admitted on April 26. His medical history included a histamine-induced angioedema treated with systemic corticosteroid therapy. Patient 2 shared patient 1's room from April 26 to April 29. On May 8, patient 2 presented with asthenia, myalgia, and cough. On May 12, he was intubated and transferred to the intensive care unit of the Lille University teaching hospital. ECMO was implemented with on May 14 for refractory hypoxemia despite optimal treatment. The patient was successfully weaned off ECMO on June 17. On July 2, percutaneous tracheotomy was performed. Currently, the patient is able to breathe spontaneously 12 hours per day but requires 3 days intermittent haemodialysis per week.

**Figure 1:**
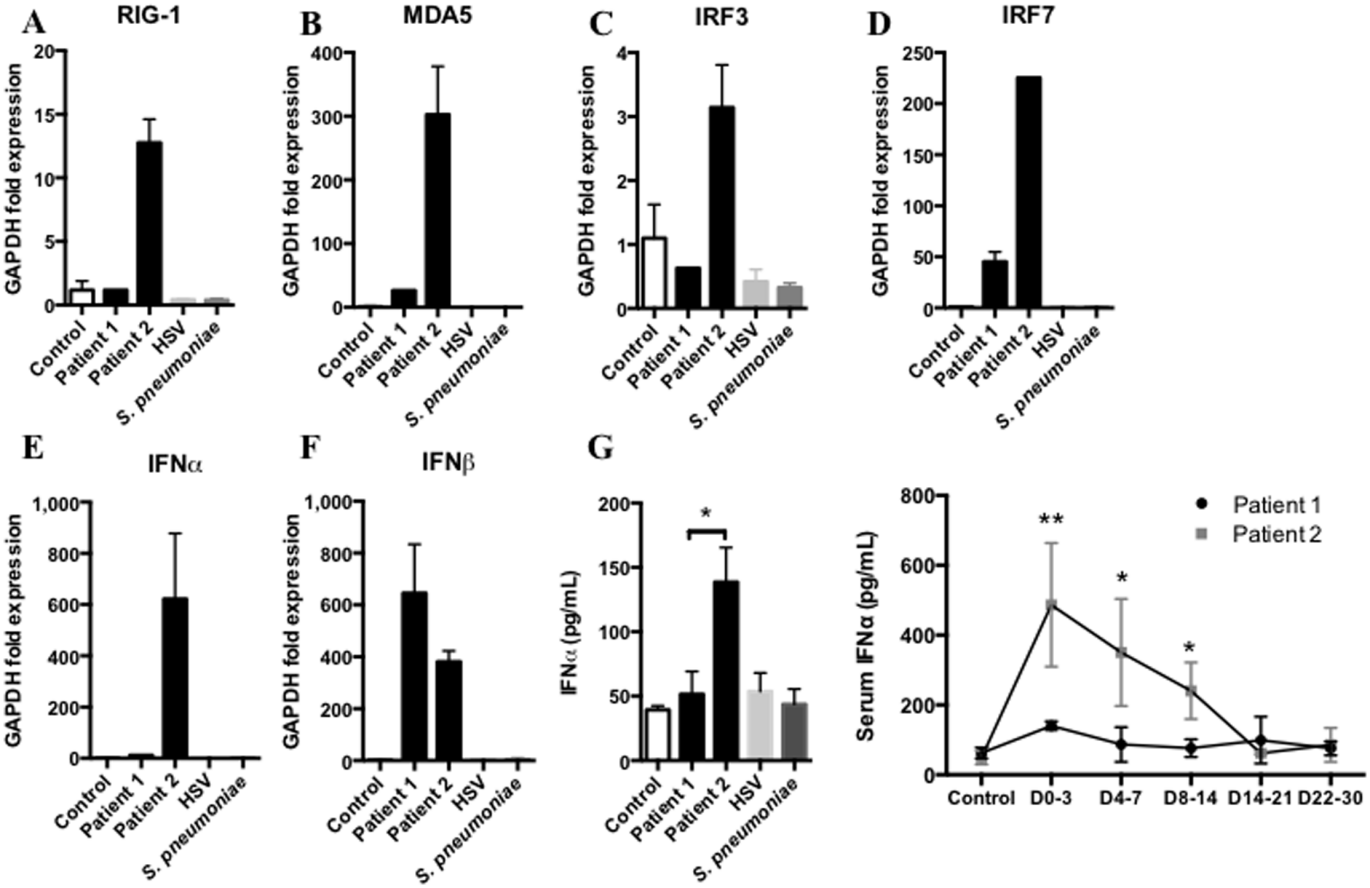
Innate immune response against MERS-CoV requires IFNα expression. **A,B,C,D,E,F** qRTPCR analysis of RIG-1, MDA-5 IRF3, IRF7, IFNα and IFNβ from first week broncho-alveolar lavage (BAL) cells of MERS-CoV infected patients, patient 1 and patient 2 (black columns), Control (white column), *S. pneumoniae*-induced pneumonia (dark grey column) and patient 2 Herpes simplex virus-induced pneumonia (light grey column) (n = 2 to 4, duplicates). **G** Assessment of IFNα protein in BAL supernatants from the first week following onset of clinical symptoms in samples from patient 1 and patient 2. Control group, HSV and bacterial samples from BALs of patients without infection, with lung HSV replication and with upper respiratory tract infection respectively. (n = 2 to 4, duplicates), and assessment of IFNα protein in sera of the 30 days following MERS-CoV infection in patient 1 (black line) and patient 2 (grey line). (n = 2 to 4, duplicates). Measurement obtained between D0–3: day 0 and day 3, D4–7: day 4 and 7, D8–14: day 8 and 14, D15–21: day 15 and 21, D22–30: day 22 and 30.

We analysed the expression of the main receptors and regulators involved in the recognition of double stranded RNA (dsRNA) from *Coronaviridae* in the cells isolated from the initial BAL samples of patients 1 and 2. Day 0 is the onset of clinical symptoms compatible with MERS-CoV infection (fever, rigors, chills) for each patient. Here we show that patient 1 could not induce the expression of the key receptors, RIG-1 and MDA-5 (Figure 1A and 1B), in response to MERS-CoV infection. Moreover, IRF3 and IRF7 expression were decreased (Figure 1C and 1D) leading to a dramatically decreased IFNα expression (Figure 1E), without any difference in IFNβ expression (Figure 1F). IFNα secretion in BAL supernatants and serum was consistent with the previous findings, IFNα levels were decreased in sera and BAL from patient 1 compared to patient 2 (Figure 1G). None of these markers were activated in control, HSV and *Streptococcus pneumoniae* infections.

**Figure 2:**
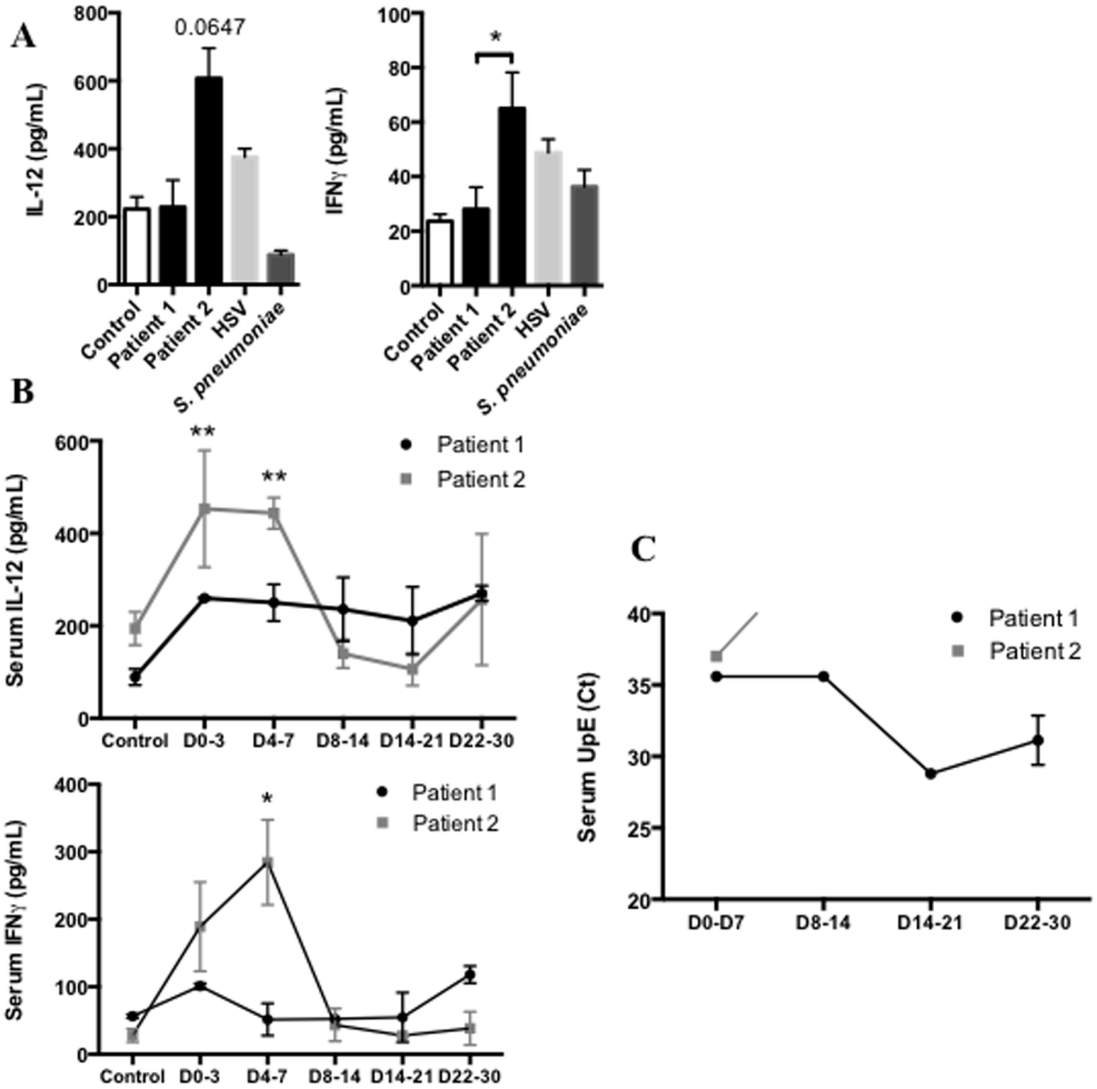
Adaptive response to control MERS-CoV replication requires early IL-12/IFNγ secretion. **A** Assessment of IL-12 and IFNγ protein in BAL supernatants of first week following onset of clinical symptoms of patient 1 and patient 2. Control group, HSV and bacterial were same samples as previously described. (n = 2 to 4, duplicates). **B** Assessment of IL-12 and IFNγ protein in sera of the 30 days following MERS-CoV infection in Patient 1 (black line) and Patient 2 (grey line). (n = 2 to 4, duplicates). **C** Assessment of MERS-CoV viral load by quantitative PCR in threshold Cycle (Ct) in serum. Measurement obtained between D0–3: day 0 and day 3, D4–7: day 4 and 7, D8–14: day 8 and 14, D15–21: day 15 and 21, D22–30: day 22 and 30.

Viral stimulation of pulmonary antigen presenting cells such as alveolar macrophages or dendritic cells leads to the secretion of IL-12 and contributes to activate T lymphocytes into CD4+ T helper-1 (Th1) lymphocytes. Recognition by IL-12R on T CD4+ Th1 cells promotes secretion of IFNγ that contributes to the early immune response by inducing death of infected cells and activating two key cells involved in viral clearance, CD8+ T effectors and Natural Killer (NK) cells[9], [13]. Here we report that IL-12 and IFNγ levels were decreased in BAL supernatants of patient 1 compared to patient 2 (Figure 2A). Furthermore, the kinetic analysis of IL-12 and IFNγ levels in the sera after infection shows that patient 2 promoted an early Th1 response mediated by IL-12 and IFNγ in response to MERS-CoV (Figure 2B). Conversely, patient 1 did not increase IL-12 and IFNγ in BAL or sera.

Interestingly, the analysis of MERS-CoV load in the serum by quantitative PCR shows that while patient 2 rapidly cleared the virus in this compartment, the virus remains detectable in the serum of patient 1 until death (Figure 2C).

**Figure 3:**
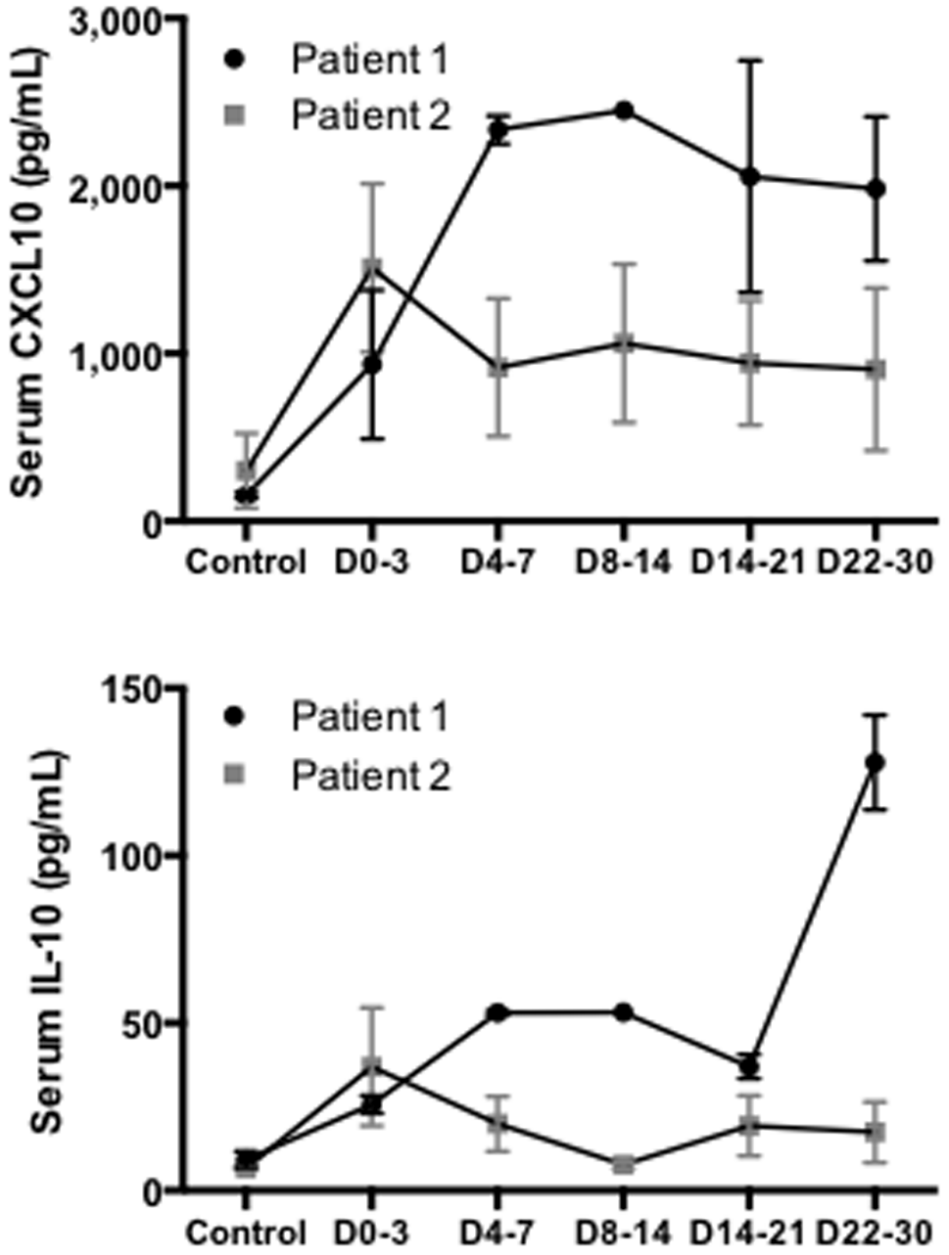
Persistent increased levels of CXCL10 and IL-10 are found in the patient with poor outcome during MERS-CoV infection. Assessment of CXCL10 and IL-10 protein in sera of the 30 days following MERS-CoV infection in patient 1 (black line) and patient 2 (grey line). (n = 2 to 4, duplicates). Measurement obtained between D0–3: day 0 and day 3, D4–7: day 4 and 7, D8–14: day 8 and 14, D15–21: day 15 and 21, D22–30: day 22 and 30.

We also measured serum levels of CXCL10, an epithelial chemokine, and IL-10, since these two cytokines were associated with a poor outcome during viral infection and particularly SARS-CoV acute respiratory infection[7], [14]–[16]. In fact, poor outcome was correlated with uncontrolled and durable IL-10 and CXCL10 secretion[7], [14]. Consistent with these findings, we found that both patients initially promoted IL-10 and CXCL10 secretion in response to MERS-CoV (Figure 3). However, while for patient 2 the response was transient for both IL-10 and CXCL10 during the first week following the onset of the first symptoms, patient 1 had a persistent increase of IL-10 and CXCL10 levels.

**Figure 4:**
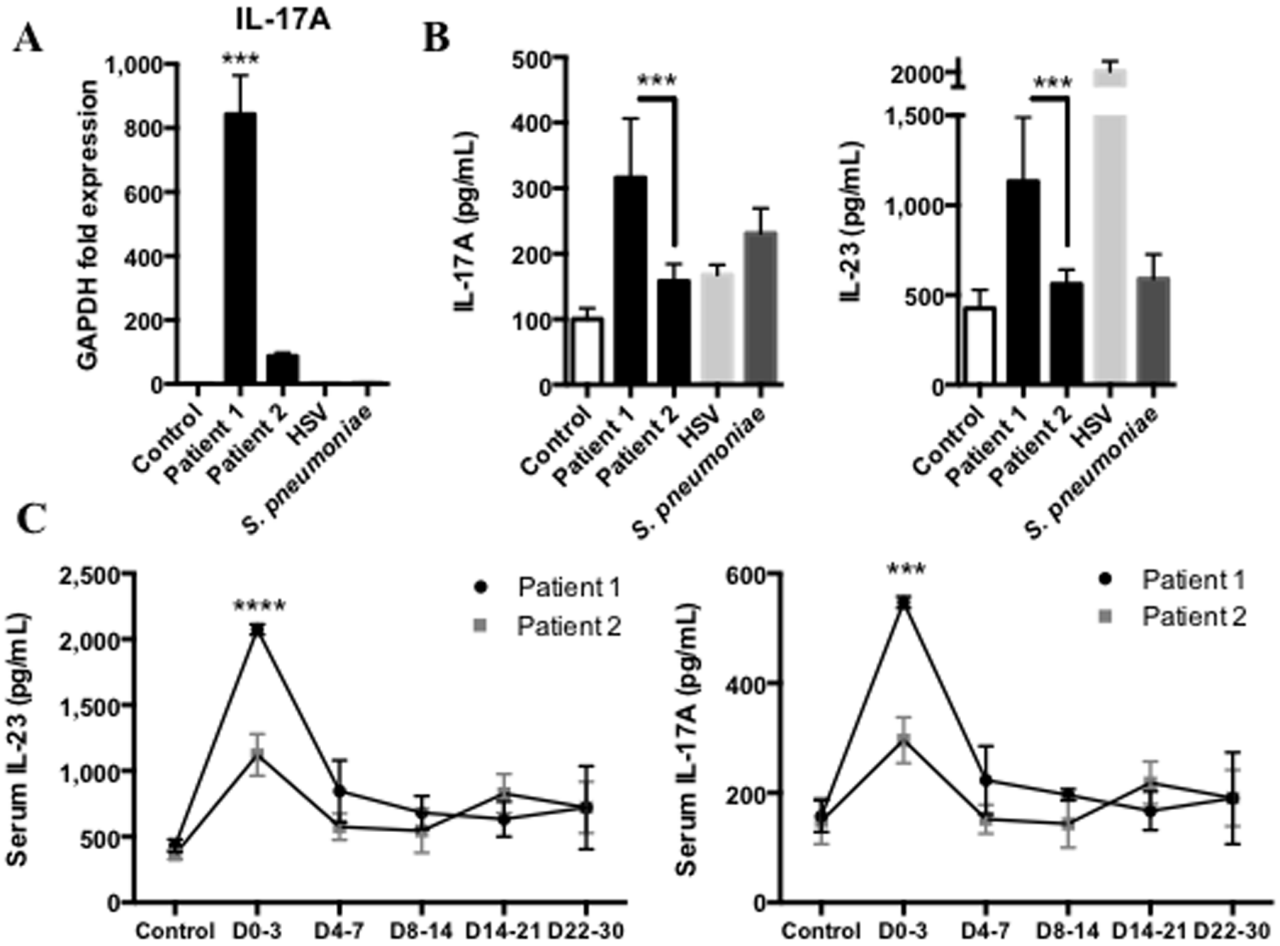
MERS-CoV induces IL-17 secretion. **A**, qRTPCR analysis of IL-17A from first week broncho-alveolar lavage (BAL) cells of MERS-CoV infected patients, Patient 1 and Patient 2 (black columns), Control (white column), *S. Pneumoniae*-induced pneumonia (dark grey column) and Patient 2 Herpes simplex virus-induced pneumonia (light grey column) (n = 2 to 4, duplicates). **B**, Assessment of IL-17A and IL-23 protein in BAL supernatants of first week following onset of clinical symptoms of patient 1 and patient 2. Control group, HSV and bacterial were same samples as previously described. (n = 2 to 4, duplicates) **C**, Assessment of IL-17A and IL-23 protein in sera of the 30 days following MERS-CoV infection in patient 1 (black line) and patient 2 (grey line). (n = 2 to 4, duplicates). Measurement obtained between D0–3: day 0 and day 3, D4–7: day 4 and 7, D8–14: day 8 and 14, D15–21: day 15 and 21, D22–30: day 22 and 30.

In a recent study, Josset *et al.* demonstrated, in an *in vitro* microarray analysis, that MERS-CoV could promote IL-17A expression compared to SARS-CoV[17]. Consistent with these findings, we report that MERS-CoV increases the expression of IL-17A in the lungs (Figure 4A). We assessed the secretion of IL-17A in BAL supernatants and showed that IL-17A secretion was increased in both patients in response to MERS-CoV (Figure 4B), with higher level for patient 1 compared to patient 2. We hypothesized the involvement of IL-23 secretion, known in humans to trigger IL-17 secretion in response to viruses[18], [19]. To this end, we assessed IL-23 secretion in the lungs and found that IL-23 secretion was significantly increased in BAL from patient 1 compared to patient 2 (Figure 4B). Finally, we found elevated IL-23 and IL-17A levels in the serum of patient 1, in the first days following the infection by MERS-CoV (Figure 4C). Interestingly, IL-23 secretion was increased in the BAL supernatant of patient 2 during HSV replication.

## Discussion

In June 2012, Zaki *et al.* reported for the first time a case of infection with Middle-East Respiratory Syndrome coronavirus (MERS-CoV)[2]. Since then, several reports have been published providing a more detailed description of the disease. Among the many questions that remain to be answered, options for treatment are a major concern for the medical community. Although it is very difficult to draw a simple line from a clinical observation to a therapeutic protocol, the analysis of the host response is a cornerstone in the design of relevant approaches. This report characterizes the immune response of two patients with different outcomes and highlights the critical role of IFNα in the innate immune response to orchestrate an early adaptive Th-1 response, mediated by IL-12 and IFNγ, against MERS-CoV infection.

Previous studies reported the critical role of IFNα, orchestrating the early immune response against viral infection, such as SARS-CoV[1], [7], [15], by up-regulating the expression of MHC class I and II[4]. In our study we show in patient 1, who presented a poor outcome, a significant decrease in receptors and regulators involved in the recognition of MERS-CoV such as RIG-1, MDA5, and IRF3-7. The decrease in IRF3 and 7 was associated with a major decrease in IFNα expression and secretion in BAL as well as in serum. Josset *et al.* demonstrated in an *in vitro* microarray analysis that MERS-CoV, like SARS-CoV[7], suppressed antigen presentation pathways by down-regulating type I and II major histocompatibility complex (MHC) genes[17]. To assess the consequences of this down-regulation, we quantified the production of two key cytokines, IL-12 and IFNγ, involved in the activation of lymphocytes into T CD4+ Th-1 cells. Our results show a clear difference between the two patients with a significant increase of both IL-12 and IFNγ in patient 2. The analysis of viral clearance shows that patient 2 rapidly cleared the infection with negative blood samples at the end of the first week, while MERS-CoV was detected in serum from patient 1 until death.

Although it is impossible to conclude on the basis of these preliminary data, it can be suggested that the IFN response, consistent with the medical literature[1], is a major component of successful anti-viral host response.

Previous studies focusing on the outcome of SARS-CoV infected patients showed that an early and robust innate immune response mediated by type-1 IFN led to high level of IFN-stimulated cytokines and was required for the development of an effective adaptive immune response[14]. Further supporting our observation, patient 1 exhibited persistent viral replication with high levels of CXCL10, a type-1 IFN triggered chemokine[14]. Conversely, patients who survived could promote type-1 IFN response, control viral replication, and down-regulate this response, exhibiting low levels of CXCL10 when recovering from infection. IL-10, a cytokine associated with Th-2 adaptive immune response that represses IFNγ secretion[20], was associated with poor outcome in the early stage of SARS-CoV infection[7]. Here we observed that patient 1 showed a persistent IL-10 secretion instead of antiviral Th-1 immune response that may have contributed to poor outcome.

Finally, Josset *et al.* previously showed that MERS-CoV could promote IL-17 expression in vitro[17]. We confirmed this observation in the lungs and sera of patient 1, and to a lesser extent in patient 2. Recently, a study reported the critical role of IRF3 in CD8+ T lymphocytes to promote IL-17 secretion. Indeed, IRF3 directly interacts with RORγt (RAR-related orphan receptor gamma) and avoids the activation of the IL-17 promoter and the Th-17 differentiation of the T CD8+ lymphocytes[21]. Here we show that patient 1 did not express IRF3 in response to MERS-CoV and secreted low levels of IFNα but high levels of IL-17, in the lungs and serum. Conversely, patient 2 expressed high levels of IRF3 (Figure 1C) that triggered IFNα secretion in the lungs, but IL-17 secretion was decreased. IRF3 in humans exhibits high polymorphism[22] that may explain the difference between immune responses and outcomes of patients 1 and 2. Further investigations may be required and the sequencing of this key protein in our two patients may contribute to better understand host susceptibility to MERS-CoV infection.

## Conclusion

**Figure 5:**
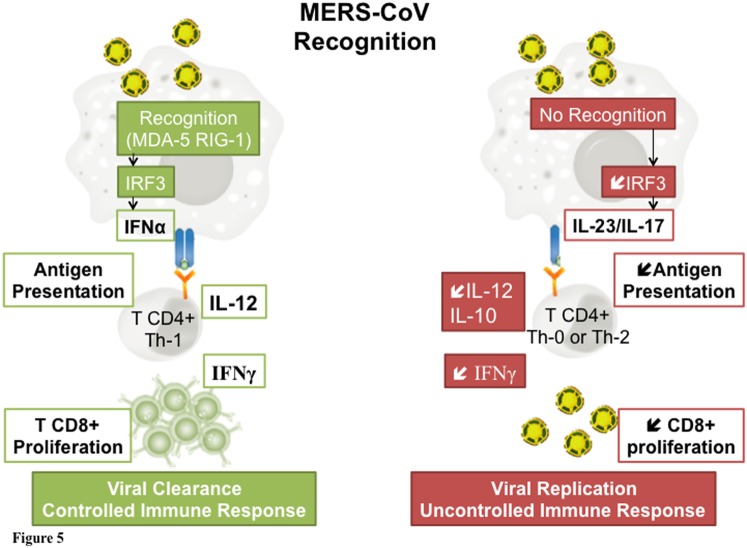
Schematic representation of key responses to MERS-CoV related to outcome. Left panel: Coronavirus are recognized by the immune system through the activation of cytosolic and membranous pattern recognition receptors (MDA-5 and RIG-1). This activation triggers IRF 3 which leads to the production of IFNα. IFN activates anti viral effectors like T CD8+ cells allowing viral clearance. Right panel: In the absence of recognition, the decrease in IRF 3 is associated to a decreased production of IL 12 and IFNγ. IL 10 production further represses IFNγ secretion leading to a decreased CD8 T lymphocytes proliferation and an increased viral replication.

Based on the analysis of the clinical course of two patients with completely different outcome, we emphasize the major role of the host immune response in MERS-CoV infection. Our results suggest a key role of the interferon pathway. From this hypothesis, a treatment with alpha interferon represents an attractive pathophysiologic approach in this disease. Several authors suggest that coronaviruses could evade the host response and blunt the interferon pathway. Patients able to trigger IFN production could optimize viral clearance and develop a controlled immune response, while the absence of pathogen recognition could lead to viral replication and uncontrolled immune response (Figure 5). Administration of interferon could therefore enhance the host response. This molecule has been suggested in the recent review published by Momattin et al.[23].
